# Data fitting and optimal control strategies for HBV acute patient cases in the United States

**DOI:** 10.1016/j.idm.2025.02.004

**Published:** 2025-02-10

**Authors:** Xuebing Chen, Yong Li, Nurbek Azimaqin, Yan Wu, Changlei Tan, Xuyue Duan, Yiyi Yuan

**Affiliations:** a School of Information and Mathematics, Yangtze University, Jingzhou, Hubei, 434023, China; b College of Mathematics and Physics, Xinjiang Agricultural University, Urumqi, 830052, China; c Institute of Operations Research and Information Engineering, Beijing University of Technology, Beijing, 100124, China; d Information Engineering College, Hunan Applied Technology University, Changde, 415100, China; e Yangtze River Engineering Vocational College, Jingzhou, Hubei, 434023, China; f Viterbi School of Engineering, University of Southern California, Los Angeles, CA, 90 0 07, USA

**Keywords:** HBV, Infection-age, Optimal control, Cost-effectiveness, Data fitting

## Abstract

Infection with Hepatitis B Virus (HBV) has been a serious public health issue worldwide. It caused more than one million fatalities per year. The mathematical modelling of the disease allows better understanding of the transmission of the disease and help the government policy makers to choose the best control strategies. With this inspiration, we proposed a novel dynamic model by incorporating infection-age structure to imitate the transmission of HBV, especially the age heterogeneity in horizontal and vertical (mother-to-child) transmission modes. We also discussed its impact on control measures and analyzed the dynamics of waning immunity and reinfection. We conducted sensitivity analysis to evaluate the effectiveness of each control measure. Our research concentrates on HBV acute patient cases in the United States data from Centre for Disease Control and Prevention (CDC). Our findings show that a mixed approach by including vaccination, medication and periodic health assessments can effectively control HBV transmission. Among these measures, we found that early vaccination with a single-dose vaccine of US$50 is the most cost-effective control strategy.

## 1 Introduction

Hepatitis B is a disease caused by hepatitis B virus (HBV), it causes liver inflammation and infection and highly contagious. It can be spread through contact with infected blood or bodily fluids or mother-to-child. In 2022, the World Health Organization (WHO) estimates that there are approximately 254 million individuals worldwide living with chronic hepatitis B. This disease causes approximately 1.2 million new infections and 1.1 million fatalities per year (Hepatitis, 2024a, 2024b, 2024c). WHO has set up an ambitious mission to eliminating viral hepatitis as a public health hazard by 2030 (Global HIV and Hepatitis and STIs Programmes (HHS), 2022).

Patients with hepatitis B can be categorized into those with acute infection and chronic carriers. Acute infection is typically the result of horizontal transmission, which may occur through sexual contact between adults, unsafe medical injections or exposure to contaminated sharp instruments. In the United States, the majority of hepatitis B is attributed to activities such as tattooing, intravenous drug use or sexual contact (Screening and Testing for Hepatitis, 2023, p. 2024). Chronic carriers are infected via vertical transmission from the mother during childbirth, which may occur through the placenta, vagina delivery, or breastfeeding (Hepatitis, 2024a, 2024b, 2024c). Chronic infection with HBV can ultimately result in severe complications such as cirrhosis and hepatocellular carcinoma, which are major causes of mortality among affected individuals.

The incubation period for HBV typically ranges from 60 to 150 days. Following this period, some individuals may develop acute symptoms, including nausea, vomiting, abdominal pain and in severe cases, acute hepatitis leading to liver failure (Symptoms of Hepatitis, 2024a, 2024b, 2024c). More than 90% of infected adults can achieve spontaneous recovery with appropriate treatment. A small proportion of patients become lifelong carriers of the virus, i.e. chronic carriers (Clinical Overview of Perinatal Hepatitis, 2024, Hepatitis B, 2024, Hepatitis B Basics, 2024, Hepatitis B Perinatal Vaccine Information, 2024). For patients with chronic hepatitis B, the progression of the disease can be effectively managed through the administration of oral antiviral medications such as Tenofovir or Entecavir, thereby delaying its deterioration (Treatment of Hepatitis, 2024a, 2024b, 2024c). The most effective method to prevent hepatitis B is through vaccination with the hepatitis B vaccine. The three-doses vaccine package of the hepatitis B can achieve a protective efficacy of over 95% against hepatitis B for more than two decades and potentially provide lifelong protection (Hepatitis, 2024a, 2024b, 2024c). Hepatitis B vaccination constitutes a critical component of comprehensive hepatitis B prevention strategies. The Centers for Disease Control and Prevention (CDC) recommends proactive vaccination for all neonates as well as adults who possess risk factors associated with hepatitis B (Hepatitis B Vaccine, 2024).

Since hepatitis B remains a significant public health concern due to its high incidence and high fatalities (both figures are over 1 million per year), we need to control or eliminate hepatitis B as soon as possible. Researchers have proposed many types of epidemiological models to investigate the transmission dynamics of the hepatitis B virus by considering specific characteristics of HBV transmission (Din et al., 2020; Ducrot et al., 2010; Edmunds et al., 1999; Greenhalgh & Rozins, 2021; Herz et al., 1996; Khan & Zaman, 2016; Nowak et al., 1996; Overton et al., 2020; O'Leary et al., 2010; Pang et al., 2010; Thornley et al., 2008; Wang et al., 2008, 2024; Wang & Wang, 2007; Yang et al., 2010; Zhang & Zhou, 2014; Zou, Zhang, & Ruan, 2010). Angela et al. (McLean & Blumberg, 1994) introduced a novel age and sex-stratified model designed for countries with high HBV transmission rates. They explored to apply the model in assessing mass vaccination strategies. Edmunds et al. (Edmunds et al., 1996) conducted a study based on hepatitis B cases in the Gambia, their model included three primary modes of transmission: perinatal, horizontal and sexual, their model incorporated age-dependent parameters to analyze the heterogeneity associated with age structure and transmission routes. Williams et al. (Williams et al., 1996) proposed a strategy for managing Hepatitis B in the UK. They provided a robust framework for evaluating the costs and benefits associated with immunization programs. Zhao et al. (Zhao et al., 2000) showed that increasing vaccination coverage could eliminate hepatitis B in China within a generation by vaccinating all infants, especially those in poor rural areas. Zou et al. (Zou, Ruan, & Zhang, 2010) categorized the host population into six compartments based on three age group and developed a dynamic model of HBV transmission by incorporating age structure. They introduced a vaccination compartment and included vertical transmission. Zhang et al. (Zhang & Xu, 2016) studied the effects of different infectivity on HBV transmission dynamics and proposed a coupled model with infection-age.

Many researchers have applied optimal control theory to investigate their HBV models and control strategies. Kamyad et al. (Kamyad et al., 2014) demonstrated that HBV infection could be effectively controlled through vaccination and treatment interventions. They formulated an appropriate optimal control objective by incorporating time-dependent control measures. Their findings indicated that a combined approach of vaccination and treatment is the most effective strategy. Din et al. (Din et al., 2021) demonstrated the existence of solution for their HBV control objective. A few researchers have utilized various control variables including isolation, treatment and vaccination to choose control strategies which is to minimize number of infected patients and associated fatalities while maximizing recovery rates (Jia et al., 2022; Jung et al., 2009; Kamyad et al., 2014; Khan et al., 2017; Laarabi et al., 2012; Lowden et al., 2014; Prosper et al., 2014). Djidjou et al. (Djidjou Demasse et al., 2016) applied optimal control theory to conduct a cost-effectiveness analysis with three intervention strategies to minimize HBV-related mortality. Finally, the optimal control measures were determined through a comparative analysis of the costs associated with different interventions (Li et al., 2016; Okosun et al., 2013; Shen et al., 2018).

As hepatitis B virus infection exhibits age-related heterogeneity, we proposed a novel dynamic model by incorporated age-structure. Given that most chronic hepatitis B carriers are infected during the perinatal period, we mainly considered the horizontal transmission mode and mother-to-child transmission mode. Here, we assume that horizontal transmission includes sexual transmission. Previous studies assumed lifelong immunity for individuals who survived hepatitis B infection (Din et al., 2020; Edmunds et al., 1999; Khan & Zaman, 2016; O'Leary et al., 2010; Pang et al., 2010; Thornley et al., 2008; Wang et al., 2008; Wang & Wang, 2007; Yang et al., 2010; Zhang & Zhou, 2014; Zou, Zhang, & Ruan, 2010). As the immunity for vaccinated individuals and recovered patients diminishes over time, we considered the reinfection of hepatitis B patients in our model. Our study concentrates on the following issues.

• Can we integrate various control measures into a model if one control variable alone is not able to control the spread of the disease?
• How to choose the most cost-effective control strategy when multiple control measures exist?

The remaining structure of this paper is organized as follows: In section 2, we develop a coupled mathematical model with infection-age combined with partial differential equations (PDE) and ordinary differential equations (ODE) to describe the dynamics of infection, treatment and vaccination in hepatitis B patients. In Section 3, we explain the meaning and range of each parameter and simulate the actual data of acute HBV patients from 2013 to 2021. Sensitivity analysis is carried out on the model in section 4. Section 5 combines the objective function and the control system to get the desired Hamiltonian function and uses Pontryagin's Maximum Principle to get the optimal control conditions. In Section 6, we study the effects of several combinations measures and find the most cost-effective strategy combination. We end this paper with some discussions and conclusions.

## 2 The HBV model

### 2.1 Model formulation

**Fig. 1:**
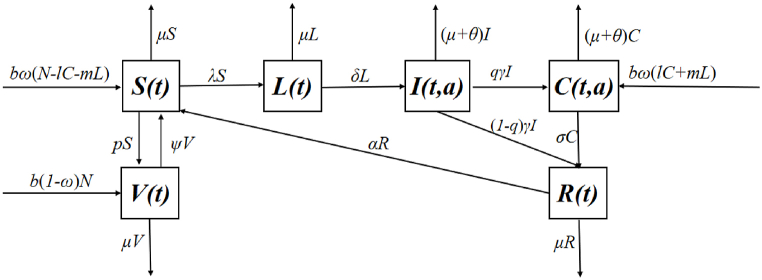
Flow chart of HBV transmission.

This section introduces an infection-age structure model for the epidemiology of hepatitis B. At time t, our model divides the population into six compartments: susceptible individuals (*S*(*t*)), latent individuals (*L*(*t*)) (those infected but asymptomatic), recovered individuals (*R*(*t*)), vaccinated individuals (*V*(*t*)), acute patients (*I*(*t*, *a*)) and chronic carriers (*C*(*t*, *a*)) where *a* is the infection-age. We assume that newborns who do not receive the hepatitis B vaccine at birth become the susceptible population *S*(*t*), they can achieve immunization if vaccinated later (Hepatitis B Perinatal Vaccine Information, 2024). Susceptible individuals (*S*(*t*)) will undergo an incubation period if infected by hepatitis B virus. Notably, during the incubation period, some infected individuals do not exhibit any symptoms, and $\lambda =\int_{0}^{\tau }\beta \left(a\right)I\left(t,a\right)da+\int_{\tau }^{\infty }\rho \beta \left(a\right)C\left(t,a\right)da$ stands for incidence. After the incubation, acute patients show some symptoms of hepatitis B, some of them may become recovered (*R*(*t*)) by taking medicine, others become chronic carriers and require lifelong medication. Chronic carriers consist of two groups: one is from a small proportion of patients who fail to recover from acute infection following treatment, the other is from infants who are infected via mother-to-child transmission during the perinatal period (see Fig. 1). Therefore, our model incorporates these two main transmission routes: perinatal transmission and horizontal transmission, we propose the following age-structured model:

$$\left\{\begin{array}{ll} \frac{dS\left(t\right)}{dt}= & b\omega N\left(t\right)-b\omega \int_{\tau }^{\infty }l\left(a\right)C\left(t,a\right)da-b\omega mL\left(t\right)-\left(\mu +p\right)S\left(t\right)+\alpha R\left(t\right)\\ & +\psi V\left(t\right)-S\left(t\right)\left(\int_{0}^{\tau }\beta \left(a\right)I\left(t,a\right)da+\int_{\tau }^{\infty }\rho \beta \left(a\right)C\left(t,a\right)da\right),\\ \frac{dL\left(t\right)}{dt}= & S\left(t\right)\left(\int_{0}^{\tau }\beta \left(a\right)I\left(t,a\right)da+\int_{\tau }^{\infty }\rho \beta \left(a\right)C\left(t,a\right)da\right)-\left(\mu +\delta \right)L\left(t\right),\\ \frac{dR\left(t\right)}{dt}= & \int_{0}^{\tau }\left(1-q\left(a\right)\right)\gamma \left(a\right)I\left(t,a\right)da+\int_{\tau }^{\infty }\sigma \left(a\right)C\left(t,a\right)da-\left(\mu +\alpha \right)R\left(t\right),\\ \frac{dV\left(t\right)}{dt}= & b\left(1-\omega \right)N\left(t\right)+pS\left(t\right)-\left(\psi +\mu \right)V\left(t\right),\\ \frac{\partial I\left(t,a\right)}{\partial t} & +\frac{\partial I\left(t,a\right)}{\partial a}\;\;=-\left(\mu +\gamma \left(a\right)+\theta \left(a\right)\right)I\left(t,a\right),\;0<a<\tau ,\\ \frac{\partial C\left(t,a\right)}{\partial t} & +\frac{\partial C\left(t,a\right)}{\partial a}=-\left(\mu +\sigma \left(a\right)+\theta \left(a\right)\right)C\left(t,a\right),\;\tau <a<\infty , \end{array}\right)$$

with the boundary condition

(2.2) $$\begin{array}{ll} I\left(t,0\right) & \;=\delta L\left(t\right),\\ C\left(t,0\right) & \;=\int_{0}^{\tau }q\left(a\right)\gamma \left(a\right)I\left(t,a\right)da+b\omega \int_{\tau }^{\infty }l\left(a\right)C\left(t,a\right)da+b\omega mL\left(t\right), \end{array}$$

and the initial condition

(2.3) $$S\left(0\right)=S_{0},\;L\left(0\right)=L_{0},\;R\left(0\right)=R_{0},\;V\left(0\right)=V_{0},\;I\left(0,a\right)=I_{0}\left(a\right),\;C\left(0,a\right)=C_{0}\left(a\right).$$

Where $N\left(t\right)=S\left(t\right)+L\left(t\right)+R\left(t\right)+V\left(t\right)+\int_{0}^{\tau }I\left(t,a\right)da+\int_{\tau }^{\infty }C\left(t,a\right)da$, $I\left(t\right)=\int_{0}^{\tau }I\left(t,a\right)da$ represents the acute patient population, $C\left(t\right)=\int_{\tau }^{\infty }C\left(t,a\right)da$ indicates chronic carrier population. Symptoms of hepatitis B appear in acute infections, symptoms usually last several weeks, chronic hepatitis B is generally defined as the persistence of hepatitis for more than six months (Kumar, 2022). Therefore, we assume that the average time for acute patients to transition to chronic carriers is about 6 months, the age of infection *τ* is increased by 6 months on the basis of the age of the original acute patient, the parameters of the model (2.1) are defined in the following Table 1.

**Table 1:** Definition of various parameters in model (2.1).

**Parameter**	Definition
*a*	The age of disease in acute patients and chronic carriers
*b*	The birth rate of population
*μ*	The natural mortality rate of population
*δ*	The rate moving from latent to acute
*l*(*a*)	The perinatal ratio of infection from the carrier mothers of age *a*
*m*	The perinatal ratio of infection from the latent mothers
*γ*(*a*)	The age-dependent rate moving from acute infections to chronic carrier or recovered
*p*	The vaccination rate against HBV
*ω*	The proportion of newborns with unsuccessful vaccinated
*ρ*	Parameters that reduce the rate of transmission due to chronic carriers
*σ*(*a*)	The recovery ratio of chronic carriers
*θ*(*a*)	The mortality rate of HBV
*β*(*a*)	The age-dependent transmission coefficient
*α*	The relapse ratio of recovered
*q*(*a*)	The ratio leaving acute infection and progressing to carrier with age *a*
*ψ*	The rate of hepatitis B vaccine loss

In our article, we assume that the age-related parameters *l*, *γ*, *σ*, *θ*, *β* and *q* are non-negative, integrable, and bounded within the defined interval; the other constant parameters are non-negative. And the initial conditions are satisfied: *S*_0_, *L*_0_, *R*_0_, $V_{0}\;\in \;R_{+}$, *I*_0_(*a*), $C_{0}\left(a\right)\;\in \;L_{+}^{1}\left(0,+\infty \right)$.

In order to facilitate the calculation and expression of the formula, we introduce the following symbols to represent the algebraic expression:

$$\begin{array}{ll} \pi_{1}\left(a\right) & \;=e^{-\int_{0}^{\tau }\left(\mu +\gamma \left(s\right)+\theta \left(s\right)\right)ds},\;0<a<\tau ,\\ \pi_{2}\left(a\right) & \;=e^{-\int_{\tau }^{\infty }\left(\mu +\sigma \left(s\right)+\theta \left(s\right)\right)ds},\;\tau <a<\infty , \end{array}$$

where *π*_1_(*a*) represents the age-specific survival probability of an acute patient individual, *π*_2_(*a*) indicates the probability of age-specific survival of a chronic carrier individual. Given the boundary conditions and initial value conditions, *I*(*t*, *a*), *C*(*t*, *a*) along the characteristic lines (*t* − *a* = *const*.), we can deduce that

(2.4) $$I\left(t,a\right)=\left\{\begin{array}{l} I\left(t-a,0\right)\pi_{1}\left(a\right),\;t>a,\;a\in \left[0,\tau \right],\;\\ I_{0}\left(a-t\right)\frac{\pi_{1}\left(a\right)}{\pi_{1}\left(a-t\right)},\;t\leq a,\;a\in \left[0,\tau \right],\; \end{array}\right)$$

and

(2.5) $$C\left(t,a\right)=\left\{\begin{array}{l} C\left(\tau +t-a,\tau \right)\pi_{2}\left(a\right),\;t+\tau >a,\;a\in \left[\tau ,+\infty \right],\;\\ C_{0}\left(a-t-\tau \right)\frac{\pi_{2}\left(a\right)}{\pi_{2}\left(a-t-\tau \right)},\;t+\tau \leq a,\;a\in \left[\tau ,+\infty \right].\; \end{array}\right)$$

With the classical result of the existence of uniqueness of functional differential equations, we can judge that the differential equations in the model have a unique solution; we will express *I*(*t*, *a*) and *C*(*t*, *a*) by the expressions (2.4) and (2.5). We can judge for all non-negative initial values. Additionally, if there is *T* such that *S*(*T*) = 0 and *S*(*t*) > 0 for 0 < *t* < *T*, we can get $\frac{dS}{dT}=b\omega N\left(t\right)-b\omega \int_{\tau }^{\infty }l\left(a\right)C\left(t,a\right)da>0$, which illustrates that *S*(*t*) ≥ 0 for all *t* ≥ 0. In the same way, it can be shown that *L*(*t*) ≥ 0, *R*(*t*) ≥ 0 and *V*(*t*) ≥ 0 for all *t* ≥ 0 and all non-negative initial values.

## 3 Data fitting

### 3.1 Fitting method

We discretize the model (2.1) by using the finite difference method (Cao et al., 2023; Iannelli et al., 1996). Initialization is performed using the initial data and the first-order backward difference separation for the differential equation (Cao et al., 2023), and advanced it in the linearize prediction correction algorithm by Implicit-Euler (Iannelli et al., 1996). In this study, we divide patients with acute conditions into six age groups. The model for each age group is discretized independently. Likewise, we treat the model for chronic carriers the same as that for acute patients. Let *T* be the final time of the simulation, with a positive integer *N*_+_, such that Δ*t* = *T*/*N*_+_ is the time step parameter of the interval discretization. For *t*^*n*^ = *n*Δ*t*, 0 ≤ *n* ≤ *N*_+_, we define that the approximate value of *S*(*t*^*n*^) is *S*^*n*^, the approximate value of *L*(*t*^*n*^) is *L*^*n*^, the approximate value of *R*(*t*^*n*^) is *R*^*n*^, the approximate value of *V*(*t*^*n*^) is *V*^*n*^, the approximate value of *I*(*t*^*n*^, *a*) is $I_{a}^{n}$, the approximate value of *C*(*t*^*n*^, *a*) is $C_{a}^{n}$. The discretization process of specific functions is as follows:

(3.1) $$\begin{array}{ll} \frac{S^{\text{n}}-S^{n-1}}{\Delta t}= & b\omega N-b\omega \sum_{a=1}^{6}l_{a}C_{a}^{n}-b\omega mL^{n}-\left(\mu +p\right)S^{n}+\alpha R^{n}+\psi V^{n}\\ & -S^{n}\sum_{a=1}^{6}\beta_{a}\left(I_{a}^{n}+\rho C_{a}^{n}\right),\\ \frac{L^{\text{n}}-L^{n-1}}{\Delta t}\;= & S^{n}\sum_{a=1}^{6}\beta_{a}\left(I_{a}^{n}+\rho C_{a}^{n}\right)-\left(\mu +\delta \right)L^{\text{n}},\\ \frac{R^{\text{n}}-R^{n-1}}{\Delta t}= & \sum_{a=1}^{6}\left(1-q_{a}\right)\gamma_{a}I_{a}^{n}+\sum_{a=1}^{6}\sigma_{a}C_{a}^{n}-\left(\mu +\alpha \right)R^{n},\\ \frac{V^{\text{n}}-V^{n-1}}{\Delta t}= & b\left(1-\omega \right)N+pS_{n}-\left(\psi +\mu \right)V^{\text{n}},\\ \frac{I_{a}^{n}-I_{a}^{n-1}}{\Delta t}\;\;\;= & -\left(\mu +\gamma_{a}+\theta_{a}\right)I_{a}^{n},\\ \frac{C_{a}^{n}-C_{a}^{n-1}}{\Delta t}= & -\left(\mu +\sigma_{a}+\theta_{a}\right)C_{a}^{n}, \end{array}$$

then,

(3.2) $$\begin{array}{ll} S^{n}= & \frac{S^{n-1}+\left(b\omega N-b\omega \overset{6}{\underset{a=1}{\sum }}l_{a}C_{a}^{n}-b\omega mL^{n}+\alpha R^{n}+\psi V^{n}\right)\Delta t}{1+\left(\overset{6}{\underset{a=1}{\sum }}\beta_{a}\left(I_{a}^{n}+\rho C_{a}^{n}\right)+\mu +p\right)\Delta t},\\ L^{\text{n}}= & \frac{L^{n-1}+S^{n}\overset{6}{\underset{a=1}{\sum }}\beta_{a}\left(I_{a}^{n}+\rho C_{a}^{n}\right)\Delta t}{1+\left(\mu +\delta \right)\Delta t},\\ R^{\text{n}}= & \frac{R^{n-1}+\left(\overset{6}{\underset{a=1}{\sum }}\left(1-q_{a}\right)\gamma_{a}I_{a}^{n}+\overset{6}{\underset{a=1}{\sum }}\sigma_{a}C_{a}^{n}\right)\Delta t}{1+\left(\mu +\alpha \right)\Delta t},\\ V^{\text{n}}= & \frac{V^{n-1}+\left(b\left(1-\omega \right)N+pS_{n}\right)\Delta t}{1+\left(\psi +\mu \right)\Delta t}, \end{array}$$

(3.2) $$\begin{array}{ll} I_{a}^{n}= & \frac{I_{a}^{n-1}}{1+\Delta t\left(\mu +\gamma_{a}+\theta_{a}\right)},\\ C_{a}^{n}= & \frac{C_{a}^{n-1}}{1+\Delta t\left(\mu +\sigma_{a}+\theta_{a}\right)}, \end{array}$$

with the numerical boundary condition:

(3.3) $$\begin{array}{ll} I_{0}^{n}= & \delta L^{\text{n}}-\left(\mu +\gamma_{a}+\theta_{a}\right)I_{a}^{n}\Delta t,\\ C_{0}^{n}= & \overset{6}{\underset{a=1}{\sum }}q_{a}\gamma_{a}I_{a}^{n}+b\omega mL^{\text{n}}+b\omega \overset{6}{\underset{a=1}{\sum }}l_{a}-\left(\mu +\sigma_{a}+\theta_{a}\right)C_{a}^{n}\Delta t. \end{array}$$

### 3.2 Parameter estimation

We estimate the model parameters by using acute hepatitis B patient data in the United States from 2013 to 2021. The data was collected by the Centers for Disease Control and Prevention (CDC) in the United States (Li et al., 2020, 2022; Viral Hepatitis Surveillance, 2024). We can simulate a series of fitted values to align with the observed trends and obtain the optimal parameter values. In the following, we first discuss data sources and references for the ranges of all parameters.

(1) The efficacy of hepatitis B vaccine can last up to 10 years (Hepatitis B Vaccine, 2024). It is estimated that the annual probability of hepatitis B virus symptoms reinfection in the recovered patients is approximately 10%, we take *α* in the range of [0, 0.2].
(2) Between 2013 and 2021, the total population of the United States fluctuated between 319,375,200 and 336,997,600 (The National Vital Statistics System, 2024). During this period, the number of births varied between 3,670,500 and 4,003,800 (The National Vital Statistics System, 2024). We compute the birth rate for each of those nine years and subsequently determine the average birth rate over this nine-years period, we get *b* = 0.0117 and suppose that the birth rate *b* has a range of [0.001, 0.02].
(3) According to the MSD manual (Kumar, 2022), if an infant is not vaccinated against hepatitis B after delivery, the risk of infection for a baby born to a mother with hepatitis B during childbirth ranges from 70% to 90%. *l* indicates the probability of perinatal transmission of hepatitis B virus from chronic carrier mothers to their infants. Edmunds et al. (Edmunds et al., 1996, 1999) published a study on hepatitis B that showed the probability of perinatal transmission from mothers of chronic carriers to infants was 0.109. Williams (Williams et al., 1996) studied the probability of transmission from mothers of acute infection to infants was 0.724, compared with 0.115 for mothers who were chronic carriers. To conduct our simulation, we constrain the infection probability range for mothers with chronic infection between 0.05 and 0.2.
(4) The annual number of natural deaths in the United States ranged from 2,599,100 to 3,280,700 between 2013 and 2021 (The National Vital Statistics System, 2024). Calculating the corresponding death rate for each year, we get an average annual mortality rate of *μ* = 0.0086, thus assuming a range for *μ* of [0.001, 0.02].
(5) Individuals infected with hepatitis B typically do not exhibit symptoms during the initial stages of infection (Hepatitis B, 2024, Hepatitis B Basics, 2024, Hepatitis B Perinatal Vaccine Information, 2024). Symptoms such as nausea, fatigue, and hepatic pain may manifest between 60 and 180 days post-infection, with an incubation period ranging from two to six months (Acute hepatitis B, 2023). We assume that the conversion rate of latent patients to acute infection *δ* ranges from (Global HIV and Hepatitis and STIs Programmes (HHS), 2022; Hepatitis B, 2024, Hepatitis B Basics, 2024, Hepatitis B Perinatal Vaccine Information, 2024).
(6) The probability of chronic carriers transmitting hepatitis B to infants ranges from 0.109 to 0.215. Given that the infectivity of latent patients is lower than that of acute infection, we can reasonably assume that the infectivity of latent patients is similar to that of chronic carriers. Thus, it is estimated that the probability of a mother with latent patient transmitting the hepatitis B virus to the infant *m* falls within the ranges of [0.05, 0.2].
(7) The vaccination coverage for hepatitis B among infants born in the United States, from birth up to 35 months of age, stands at 90.5% (Hepatitis B Perinatal Vaccine Information, 2023); from the WHO, one dose of the hepatitis B vaccine induces seroprotection in approximately 30% of recipients, two doses result in seroprotection in about 90% of individuals, and a complete three-dose series achieves seroprotection rates of 95% or higher (Hepatitis: How can I protect, 2023). We posit that all infants and young children, from birth to 35 months of age, who have received the hepatitis B vaccination regimen consisting of three doses (Yusuf et al., 2000), and the probability of success of infant hepatitis B vaccination is 0.855. Therefore, it is assumed that the failure rate of infant and young child vaccination *ω* ranges from [0, 0.2].
(8) The hepatitis B vaccination coverage among susceptible individuals aged 19 years and older is 40% (Vaccination Coverage among Adults in, 2023). We assume that all the vaccinated individuals have received three doses of the vaccine, resulting in an antibody production rate exceeding 95%, so the probability of susceptible individuals becoming immune following vaccination is estimated *p* = 0.38. Let us assume that *p* has a value range of [0, 0.5].
(9) The CDC has stated that the hepatitis B vaccine dose not guarantee lifelong immunity and regular liver examinations are still necessary. For individuals who have successfully received the hepatitis B vaccine, the antibodies generated by the body typically last between 3 and 10 years. We estimate that the annual rate of antibody decline *ψ* in the average vaccinated individual ranges from [0.1, 0.34].
(10) The associated infection rate in patients with chronic carriers was 0.16 (Edmunds et al., 1996). We assume during the simulation that *ρ* has a value range of [0, 0.5].
(11) Approximately 90%–95% of acute patients can successfully clear the virus through treatment and achieve immunity (Hepatitis B Basics, 2024). Let us assume that the range of *q* is [0, 0.5].
(12) Patients who initially present with acute symptoms and subsequently experience a duration of six months or longer are reclassified as chronic carriers (Erin, 2023); let us assume that *γ* has a range of [0, 2].
(13) The highest prevalence of chronic hepatitis B cases among individuals ages 30–39 and 40–49 (Hepatitis B, 2024, Hepatitis B Basics, 2024). Generally, patients with chronic hepatitis B are lifelong carriers of the virus and must rely on pharmacological interventions and lifestyle modifications for management (Number and rate, 2022). It is assumed that chronic patients may harbor the virus for a period ranging from 30 to 40 years, the value range of *σ* is [0, 0.1].
(14) Between 2018 and 2022, the number of deaths from hepatitis B in the United States fluctuated between 1649 and 1797 (Numbers and rates, 2023), accounting for 1 in 1000 deaths; we assume that the death rate due to disease *θ* is [0, 0.00002].
(15) The age-dependent transmission coefficient is unknown, so we set $\beta \;\in \;\left(\left(0,1\right)\right]$.

**Fig. 2:**
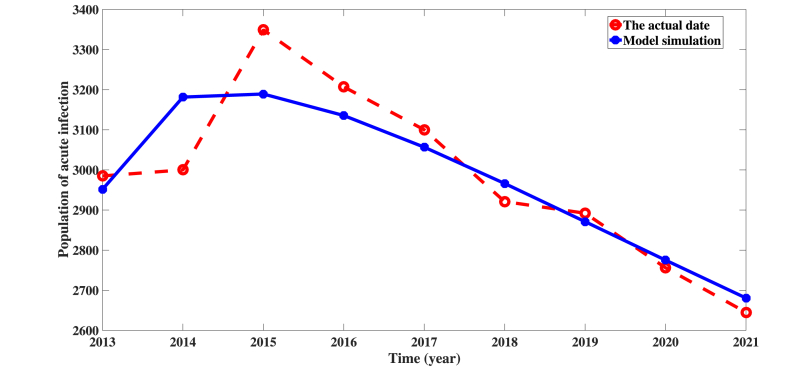
Real data and model (2.1) simulation results from acute hepatitis B patients in the United States from 2013 to 2021.

Through the model and the range of its corresponding parameters, we conduct a data fitting analysis for the number of acute patients. As shown in Fig. 2, the trend of the fitting results is consistent with the data of acute hepatitis B patients in the United States from 2013 to 2021. The best fitting parameter values shown in Table 2 can predict future trends in acute patients in the United States, and these parameter values can be used to determine sensitivity coefficients, which are described in detail in section 4. As can be seen from Fig. 2, fitting results exhibit superior accuracy and reliability, thereby providing robust validation for the model's applicability in simulating the transmission characteristics of hepatitis B.

**Table 2:** The parameters estimation of the HBV model (2.1).

Parameter	Range	Value	Unit	Sources
*α*	[0, 0.2]	0.1069	year^−1^	fitting
*b*	[0, 0.02]	0.01	year^−1^	fitting
*l*	[0.05, 0.2]	0.2	none	fitting
*μ*	[0, 0.02]	0.0086	year^−1^	fitting
*δ*	[2,6]	2	year^−1^	fitting
*m*	[0.05, 0.2]	0.0988	none	fitting
*ω*	[0, 0.2]	0.0012	none	fitting
*p*	[0, 0.5]	0.3769	year^−1^	fitting
*ψ*	[0.1, 0.34]	0.1047	year^−1^	fitting
*q*	[0, 0.5]	0.0406	none	fitting
*ρ*	[0, 0.5]	0.2	year^−1^	fitting
*γ*	[0, 2]	0.0427	year^−1^	fitting
*θ*	[0, 0.00002]	1.2837 × 10^−18^	year^−1^	fitting
*σ*	[0, 0.1]	0.001	year^−1^	fitting
*β*	[0, 1]	7.6207 × 10^−8^	none	fitting

## 4 Sensitivity analysis

**Fig. 3:**
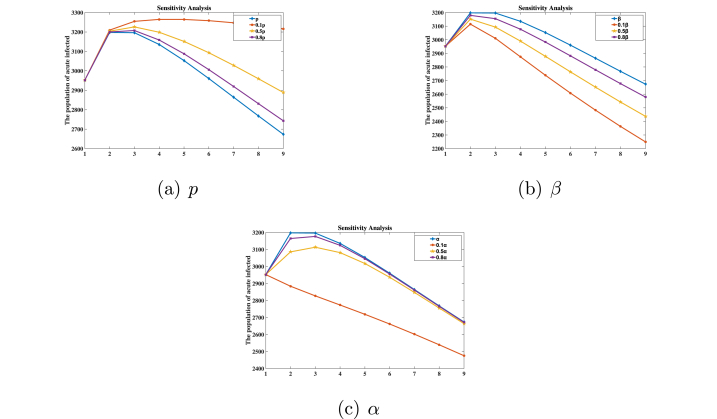
Sensitivity analysis of parameter *p*, *β*, *α*.

We can use sensitivity analysis to determine the relatively significant effects of different parameters that contribute to the hepatitis B epidemic. Due to the complex construction of this model, the basic reproduction number $R_{0}$ cannot be obtained to judge its sensitive parameters. Therefore, we judge the degree of its influence on the dynamics model by changing the size of the parameter value, especially in relation to the size of *I*(*t*). When determining the best way to control human mortality caused by epidemics, we typically run a series of tests to discover parameters that have a significant impact on the dynamic behavior of the system; the detailed process is shown in Fig. 3. As demonstrated in the experiment, when comparing parameter values at 0.1 times, 0.5 times, and 0.8 times their original values, it is observed that the number of acute patients varies correspondingly with changes in these parameters. The most significant fluctuations are noted for the parameters *p*, *β*, and *α*, as illustrated in Fig. 3.

When modifying the relevant parameters, it is observed that alterations in three specific parameters, namely *p*, *β*, and *α*, exert the most significant influence on *I*(*t*). Consequently, it can be inferred that these three parameters are the most sensitive with respect to changes in *I*(*t*).

## 5 Optimal control

### 5.1 Analysis of optimal control

A variety of strategies exist for the treatment and intervention of HBV (Kao & Chen, 2002; Maynard, 1990; Meireles et al., 2015), and we typically employ optimal control theory to obtain the optimal therapeutic intervention strategies, thereby achieving the objectives of reducing the number of susceptible and infected individuals while increasing the number of recovered and immune individuals. In the analysis of the treatment for asymptomatic HBV-infected individuals (latent patients), we assume that economic expenditures are not considered. Patients with symptomatic HBV infection require targeted therapeutic interventions to effectively manage the disease. In terms of preventive measures, we can consider measures such as vaccination of hepatitis B susceptible patients and regular liver examination of immunized people. Based on the sensitivity analysis in section 4, the three parameters of *p*, *β* and *α* have a greater impact on acute patients and chronic patients, and the corresponding three intervention strategies (also known as control) can be implemented on the initial model (2.1). Control is a time-dependent function and is constrained by the upper and lower bounds. We consider using *h*_1_(*t*) to increase the hepatitis B vaccine coverage rate (*p*) in susceptible populations, where the rate of the hepatitis B vaccination strategy for susceptible patients at time *t* is *h*_1_(*t*). Second, the associated force of transmission rate of susceptible person (*β*) is reduced by factors of *h*_2_(*t*), where *h*_2_(*t*) shows preventive measures such as spatial isolation and the rate of contact reduction strategies for infected persons at the time *t* is *h*_2_(*t*). Then, *h*_3_ is to increase the duration of immunity and reduce the risk of reinfection in recovered patients (*α*) at time *t* (such as regular screening and liver tests are performed).

We consider the above assumptions and the introduction of the three controls and use the parameters associated with the model (2.1) and Table 2; the following control system is obtained:

$$\left\{\begin{array}{ll} \frac{dS\left(t\right)}{dt}= & b\omega N\left(t\right)-b\omega q_{1}\left(t\right)-b\omega mL\left(t\right)-q_{2}\left(t\right)\left(1-h_{2}\left(t\right)\right)S\left(t\right)-\mu S\left(t\right)\\ & +\alpha \left(1-h_{3}\left(t\right)\right)R\left(t\right)-p\left(1+h_{1}\left(t\right)\right)S\left(t\right)+\psi V\left(t\right),\\ \frac{dL\left(t\right)}{dt}= & q_{2}\left(t\right)\left(1-h_{2}\left(t\right)\right)S\left(t\right)-\left(\mu +\delta \right)L\left(t\right),\\ \frac{dR\left(t\right)}{dt}= & q_{3}\left(t\right)+q_{4}\left(t\right)-\mu R\left(t\right)-\alpha \left(1-h_{3}\left(t\right)\right)R\left(t\right),\\ \frac{dV\left(t\right)}{dt}= & bN\left(t\right)-b\omega N\left(t\right)+p\left(1+h_{1}\left(t\right)\right)S\left(t\right)-\left(\psi +\mu \right)V\left(t\right),\\ \frac{\partial I\left(t,a\right)}{\partial t} & +\frac{\partial I\left(t,a\right)}{\partial a}\;\;=-\left(\mu +\gamma \left(a\right)+\theta \left(a\right)\right)I\left(t,a\right),\\ \frac{\partial C\left(t,a\right)}{\partial t} & +\frac{\partial C\left(t,a\right)}{\partial a}=-\left(\mu +\sigma \left(a\right)+\theta \left(a\right)\right)C\left(t,a\right),\\ I\left(t,0\right) & =\delta L\left(t\right),\;\;\;C\left(t,0\right)=q_{5}\left(t\right)+b\omega mL\left(t\right)+b\omega q_{1}\left(t\right), \end{array}\right)$$

where

$$\begin{array}{c} q_{1}\left(t\right)=\int_{\tau }^{\infty }l\left(a\right)C\left(t,a\right)da,\;q_{2}\left(t\right)=\int_{0}^{\tau }\beta \left(a\right)I\left(t,a\right)da+\int_{\tau }^{\infty }\beta \left(a\right)\rho C\left(t,a\right)da,\\ q_{3}\left(t\right)=\int_{0}^{\tau }\left(1-q\left(a\right)\right)\gamma \left(a\right)I\left(t,a\right)da,\;q_{4}\left(t\right)=\int_{\tau }^{\infty }\sigma \left(a\right)C\left(t,a\right)da,\\ q_{5}\left(t\right)=\int_{0}^{\tau }q\left(a\right)\gamma \left(a\right)I\left(t,a\right)da. \end{array}$$

We propose employing time-dependent control strategies to achieve optimal management within a constrained timeframe. Therefore, we apply Pontryagin's Maximum Principle (Pontryagin, 2018) to determine the control conditions that can achieve the spread of infectious diseases within a limited period, and each control will generate some corresponding costs. If the objective function can be minimized, the control scheme we implement will be optimal. The following objective function is assumed:

(5.2) $$\begin{array}{ll} J(h_{1}\left(t\right),h_{2}\left(t\right),h_{3}\left(t\right))\;= & \int_{0}^{T}A_{1}\delta L\left(t\right)dt+\int_{0}^{T}A_{2}b\omega mL\left(t\right)dt\\ & +\int_{0}^{T}\int_{0}^{A}A_{2}\left(q\left(a\right)\gamma \left(a\right)I\left(t,a\right)+b\omega l\left(a\right)C\left(t,a\right)\right)dadt\\ & +\int_{0}^{T}B_{1}h_{1}\left(t\right)b\omega \left(S\left(t\right)+L\left(t\right)+V\left(t\right)+R\left(t\right)-mL\left(t\right)\right)dt\\ & +\int_{0}^{T}\int_{0}^{A}B_{1}h_{1}\left(t\right)b\omega \left(I\left(t,a\right)+C\left(t,a\right)-l\left(a\right)C\left(t,a\right)\right)dadt\\ & +\int_{0}^{T}B_{2}h_{3}\left(t\right)R\left(t\right)dt\\ & +\int_{0}^{T}\frac{1}{2}\left(\lambda_{1}h_{1}^{2}\left(t\right)+\lambda_{2}h_{2}^{2}\left(t\right)+\lambda_{3}h_{3}^{2}\left(t\right)\right)dt, \end{array}$$

where [0, *T*] indicates the time range, [0, *A*] indicates the age. *A*_1_, *A*_2_, *B*_1_, *B*_2_, *λ*_1_, *λ*_2_, and *λ*_3_ are the positive weight coefficient in the objective function, see Table 3 for details. In the objective function (5.2), *bω*(*S* + *L* + *V* + *R* − *mL*) and *bω*(*I* + *C* − *lC*) represent the group of newborn infants who should be vaccinated, *bωmL* and *bωlC* represent infants and young children who have been infected through mother-to-child transmission. Because the hepatitis B vaccine is ineffective for individuals who have already been infected with or exhibited symptoms of hepatitis B (Obeagu, 2023), infants who have been infected via mother-to-child transmission at birth are not candidates for vaccination and will require subsequent pharmacological treatment.

**Table 3:** Definition of the positive weight coefficient in the objective function.

**Parameter**	Definition
*A*_1_	The per capita costs of treating acute patients and costs of isolation
*A*_2_	The per capita cost of treating chronic patients
*B*_1_	The cost of three doses of hepatitis B vaccine
*B*_2_	The per capita cost of reexamination for rehabilitated persons
*λ*_1_	The cost of artificial vaccination of hepatitis B vaccine
*λ*_2_	The cost of quarantine measures to control patient contact
*λ*_3_	The cost of manual examination for patients who have recovered to prevent reinfection

With the given objective function *J*(*h*_1_(*t*), *h*_2_(*t*), *h*_3_(*t*)), aims to minimize the number of acute infected (*I*), and achieve minimizing the cost of control *h*_1_(*t*), *h*_2_(*t*) and *h*_3_(*t*). It finds an optimal control set $h_{1}^{*}\left(t\right)$,$h_{2}^{*}\left(t\right)$ and $h_{3}^{*}\left(t\right)$.

$$\begin{array}{c} J\left(h_{1}^{*}\left(t\right),h_{2}^{*}\left(t\right),h_{3}^{*}\left(t\right)\right)=\underset{h\in U}{\min}J\left(h_{1}\left(t\right),h_{2}\left(t\right),h_{3}\left(t\right)\right), \end{array}$$

the control set is:

$$\begin{array}{c} U=\left\{\left(h_{1},h_{2},h_{3}\right)|h_{i}\left(t\right)(i=1,2,3)\;is\;measurable,\;h_{i}\left(t\right)\in \left(0,1\right),\;t\in \left[0,T\right]\right\}. \end{array}$$

### 5.2 Application of optimal control

The existence of optimal control must meet the following conditions: Firstly, the solution sets of the objective function system and the control system are non-empty. Secondly, every differential equation in the control system is linear in every control variable, *h*_*i*_(*t*)(*i* = 1, 2, 3). Thirdly, the integrand of the objective function is convex in the control set *U*. Finally, the control set *U* is closed. Roxin and Fleming (Fleming & Rishel, 2012; Roxin, 1962, 1965) show that if these conditions are met, there is an optimal control. In order to find the optimal solution, we first require the Lagrange and Hamiltonian of the optimal control system and then apply Pontryagin's Maximum Principle to solve the optimal control. We can obtain the Lagrange function from the objective function:

(5.3) $$\begin{array}{ll} L\left(t,a,h_{1},h_{2},h_{3}\right)\;= & A_{1}\delta L\left(t\right)+A_{2}b\omega mL\left(t\right)\\ & +A_{2}\left(q\left(a\right)\gamma \left(a\right)I\left(t,a\right)+b\omega l\left(a\right)C\left(t,a\right)\right)\\ & +B_{1}h_{1}\left(t\right)b\omega \left(S\left(t\right)+L\left(t\right)+V\left(t\right)+R\left(t\right)-mL\left(t\right)\right)\\ & +B_{1}h_{1}\left(t\right)b\omega \left(I\left(t,a\right)+C\left(t,a\right)-l\left(a\right)C\left(t,a\right)\right)\\ & +B_{2}h_{3}\left(t\right)R\left(t\right)\\ & +\frac{1}{2}\left(\lambda_{1}h_{1}^{2}\left(t\right)+\lambda_{2}h_{2}^{2}\left(t\right)+\lambda_{3}h_{3}^{2}\left(t\right)\right). \end{array}$$

Next, we define the Hamiltonian function of the control system:

$$\begin{array}{c} H\left(t,a,h\left(t\right),\lambda \left(t\right)\right)=L\left(t,a,h\left(t\right)\right)+\lambda \left(t\right)g\left(t,a,h\left(t\right)\right). \end{array}$$

The Hamiltonian function comprises the sum of the integrated of the objective function and the integral involving the adjoint variables and the control function. Each differential equation in the control system is multiplied by an associated adjoint variable, with each of the six chamber variables having a corresponding adjoint variable (e.g. *λ*_*S*_ corresponds to *S*). In this paper, by integrating the control system, we derive the detailed Hamiltonian function as follows:

(5.4) $$\begin{array}{ll} H\left(t,a,h_{1},h_{2},h_{3}\right)\;= & A_{1}\delta L\left(t\right)+A_{2}b\omega mL\left(t\right)+A_{2}\left(q\left(a\right)\gamma \left(a\right)I\left(t,a\right)+b\omega l\left(a\right)C\left(t,a\right)\right)\\ & B_{1}h_{1}\left(t\right)b\omega \left(S\left(t\right)+L\left(t\right)+V\left(t\right)+R\left(t\right)-mL\left(t\right)+I\left(t,a\right)+C\left(t,a\right)\right)\\ & \left(-l\left(a\right)C\left(t,a\right)\right)+\frac{1}{2}\left(\lambda_{1}h_{1}{\left(t\right)}^{2}+\lambda_{2}h_{2}{\left(t\right)}^{2}+\lambda_{3}h_{3}{\left(t\right)}^{2}\right)+B_{2}h_{3}\left(t\right)R\left(t\right)\\ & +\lambda_{S}\left(t\right)\left[b\omega N\left(t\right)-b\omega q_{1}\left(t\right)-b\omega mL\left(t\right)-q_{2}\left(t\right)\left(1-h_{2}\left(t\right)\right)S\left(t\right)\right)\\ & \left(-\mu S\left(t\right)+\alpha \left(1-h_{3}\left(t\right)\right)R\left(t\right)-p\left(1+h_{1}\left(t\right)\right)S\left(t\right)+\psi V\left(t\right)\right]\\ & +\lambda_{L}\left(t\right)\left[q_{2}\left(t\right)\left(1-h_{2}\left(t\right)\right)S\left(t\right)-\left(\mu +\delta \right)L\left(t\right)\right]\\ & +\lambda_{R}\left(t\right)\left[q_{3}\left(t\right)+q_{4}\left(t\right)-\mu R\left(t\right)-\alpha \left(1-h_{3}\left(t\right)\right)R\left(t\right)\right]\\ & +\lambda_{V}\left(t\right)\left[bN\left(t\right)-b\omega N\left(t\right)+p\left(1+h_{1}\left(t\right)\right)S\left(t\right)-\left(\psi +\mu \right)V\left(t\right)\right]\\ & +\lambda_{I}\left(t,a\right)\left[-\left(\mu +\gamma \left(a\right)+\theta \left(a\right)\right)I\left(t,a\right)\right]\\ & +\lambda_{C}\left(t,a\right)\left[-\left(\mu +\sigma \left(a\right)+\theta \left(a\right)\right)C\left(t,a\right)\right], \end{array}$$

where *λ*_*S*_, *λ*_*L*_, *λ*_*R*_, *λ*_*V*_, *λ*_*I*_ and *λ*_*C*_ is an adjoint function to be determined.

Theorem 1 *Set*$\bar{S}\left(t\right)$, $\bar{L}\left(t\right)$, $\bar{R}\left(t\right)$, $\bar{V}\left(t\right)$, $\bar{I}\left(t,a\right)$*and*$\bar{C}\left(t,a\right)$*as the state of optimal solution of optimal control*, *h*_1_(*t*), *h*_2_(*t*) *andh*_3_(*t*) *is the optimal control variables for the optimal control problem* (5.1). *Then there exist adjoint variables* *λ*_*S*_, *λ*_*L*_, *λ*_*R*_, *λ*_*V*_, *λ*_*I*_ *and* *λ*_*C*_ *that satisfy*

(5.5) $$\begin{array}{ll} \frac{d\lambda_{S}\left(t\right)}{dt}\;= & -b\omega \lambda_{S}+q_{2}\left(t\right)\left(1-h_{2}\left(t\right)\right)\lambda_{S}+\mu \lambda_{S}+p\left(1+h_{1}\left(t\right)\right)\lambda_{S}\\ & -q_{2}\left(t\right)\left(1-h_{2}\left(t\right)\right)\lambda_{L}-p\left(1+h_{1}\left(t\right)\right)\lambda_{V}-b\left(1-\omega \right)\lambda_{V}\\ & -B_{1}h_{1}\left(t\right)b\omega ,\\ \frac{d\lambda_{L}\left(t\right)}{dt}\;= & -b\omega \lambda_{S}+b\omega m\lambda_{S}+\left(\mu +\delta \right)\lambda_{L}-b\left(1-\omega \right)\lambda_{V}-A_{1}\delta \\ & -A_{2}b\omega m-B_{1}h_{1}\left(t\right)b\omega +B_{1}h_{1}\left(t\right)b\omega m,\\ \frac{d\lambda_{R}\left(t\right)}{dt}\;= & -b\omega \lambda_{S}-\alpha \left(1-h_{3}\left(t\right)\right)\lambda_{S}+\mu \lambda_{R}+\alpha \left(1-h_{3}\left(t\right)\right)\lambda_{R}\\ & -b\left(1-\omega \right)\lambda_{V}-B_{1}h_{1}\left(t\right)b\omega +B_{2}h_{3}\left(t\right),\\ \frac{d\lambda_{V}\left(t\right)}{dt}\;= & -b\omega \lambda_{S}-\psi \lambda_{S}-b\left(1-\omega \right)\lambda_{V}+\left(\psi +\mu \right)\lambda_{V}\\ & -B_{1}h_{1}\left(t\right)b\omega , \end{array}$$

$$\begin{array}{ll} \frac{\partial \lambda_{I}\left(t,a\right)}{\partial t}+\frac{\partial \lambda_{I}\left(t,a\right)}{\partial a}\;\;= & -b\omega \lambda_{S}-b\left(1-\omega \right)\lambda_{V}+\left(\mu +\gamma \left(a\right)+\theta \left(a\right)\right)\lambda_{I}\\ & -A_{2}q\left(a\right)\gamma \left(a\right)-B_{1}h_{1}\left(t\right)b\omega ,\\ \frac{\partial \lambda_{C}\left(t,a\right)}{\partial t}+\frac{\partial \lambda_{C}\left(t,a\right)}{\partial a}= & -b\omega \lambda_{S}-b\left(1-\omega \right)\lambda_{V}+\left(\mu +\sigma \left(a\right)+\theta \left(a\right)\right)\lambda_{I}\\ & -A_{2}b\omega l\left(a\right)-B_{1}h_{1}\left(t\right)b\omega \left(1-l\left(a\right)\right), \end{array}$$

*and under the transversal condition* *λ*_*j*_(*T*) = 0, (*j* = *S*, *L*, *R*, *V*, *I*, *C*), *the corresponding optimal control is given*

(5.6) $$\begin{array}{ll} h_{1}^{*}\left(t\right)= & max\left(0,min\left({\bar{h}}_{1}\left(t\right),1\right)\right),\\ h_{2}^{*}\left(t\right)= & max\left(0,min\left({\bar{h}}_{2}\left(t\right),1\right)\right),\\ h_{2}^{*}\left(t\right)= & max\left(0,min\left({\bar{h}}_{3}\left(t\right),1\right)\right), \end{array}$$

*where*,

$$\begin{array}{lll} {\bar{h}}_{1}\left(t\right) & \;= & \frac{-B_{1}b\omega \left(\bar{N}\left(t\right)-mL\left(t\right)-q_{1}\left(t\right)\right)+\lambda_{S}\left(t\right)p\bar{S}\left(t\right)-\lambda_{V}\left(t\right)p\bar{S}\left(t\right)}{\lambda_{1}},\\ {\bar{h}}_{2}\left(t\right) & \;= & \frac{-\lambda_{S}\left(t\right)q_{2}\left(t\right)\bar{S}\left(t\right)+\lambda_{L}\left(t\right)q_{2}\left(t\right)\bar{S}\left(t\right)}{\lambda_{2}},\\ {\bar{h}}_{3}\left(t\right) & \;= & \frac{\lambda_{S}\left(t\right)\alpha \bar{R}\left(t\right)-\lambda_{R}\left(t\right)\alpha \bar{R}\left(t\right)-B_{1}\bar{R}\left(t\right)}{\lambda_{3}}. \end{array}$$

Proof Using Hamiltonian functions to determine adjoint equations and transversal conditions, by taking Hamiltonian derivatives of *S*, *L*, *R*, *V*, *I*, *C*, we get

$$\begin{array}{lll} \frac{d\lambda_{S}\left(t\right)}{dt} & =\; & -\frac{\partial H}{\partial S}=\;-b\omega \lambda_{S}+q_{2}\left(t\right)\left(1-h_{2}\left(t\right)\right)\lambda_{S}+\mu \lambda_{S}\\ & & +p\left(1+h_{1}\left(t\right)\right)\lambda_{S}-q_{2}\left(t\right)\left(1-h_{2}\left(t\right)\right)\lambda_{L}-b\left(1-\omega \right)\lambda_{V}\\ & & -p\left(1+h_{1}\left(t\right)\right)\lambda_{V}-B_{1}h_{1}\left(t\right)b\omega ,\\ \frac{d\lambda_{L}\left(t\right)}{dt} & = & -\frac{\partial H}{\partial L}=\;-b\omega \lambda_{S}+b\omega m\lambda_{S}+\left(\mu +\delta \right)\lambda_{L}-b\left(1-\omega \right)\lambda_{V}\\ & & -A_{1}\delta -A_{2}b\omega m-B_{1}h_{1}\left(t\right)b\omega +B_{1}h_{1}\left(t\right)b\omega ,\\ \frac{d\lambda_{R}\left(t\right)}{dt} & = & -\frac{\partial H}{\partial R}=\;-b\omega \lambda_{S}-\alpha \left(1-h_{3}\left(t\right)\right)\lambda_{S}+\mu \lambda_{R}-b\left(1-\omega \right)\lambda_{V}\\ & & +\alpha \left(1-h_{3}\left(t\right)\right)\lambda_{R}-B_{1}h_{1}\left(t\right)b\omega ,\\ \frac{d\lambda_{V}\left(t\right)}{dt} & = & -\frac{\partial H}{\partial V}=\;-b\omega \lambda_{S}-\psi \lambda_{S}-b\left(1-\omega \right)\lambda_{V}+\left(\psi +\mu \right)\lambda_{V}, \end{array}$$

$$\begin{array}{lll} \frac{\partial \lambda_{I}\left(t,a\right)}{\partial t}+\frac{\partial \lambda_{I}\left(t,a\right)}{\partial a} & =\; & -\frac{\partial H}{\partial I}=-b\omega \lambda_{S}-b\left(1-\omega \right)\lambda_{V}+\left(\mu +\gamma \left(a\right)+\theta \left(a\right)\right)\lambda_{I}\\ & & -A_{2}q\left(a\right)\gamma \left(a\right)-B_{1}h_{1}\left(t\right)b\omega ,\\ \frac{\partial \lambda_{C}\left(t,a\right)}{\partial t}+\frac{\partial \lambda_{C}\left(t,a\right)}{\partial a} & = & -\frac{\partial H}{\partial C}=-b\omega \lambda_{S}-b\left(1-\omega \right)\lambda_{V}+\left(\mu +\sigma \left(a\right)+\theta \left(a\right)\right)\lambda_{I}\\ & & -A_{2}b\omega l\left(a\right)-B_{1}h_{1}\left(t\right)b\omega \left(1-l\left(a\right)\right). \end{array}$$

By using optimality conditions, the optimal equation is

(5.7) $$\frac{\partial H}{\partial h_{1}}=\frac{\partial H}{\partial h_{2}}=\frac{\partial H}{\partial h_{3}}=0.$$

According to the optimal equation

$$\frac{\partial H}{\partial h_{1}}=B_{1}b\omega \left(\bar{S}\left(t\right)+\bar{L}\left(t\right)+\bar{V}\left(t\right)+\bar{R}\left(t\right)-m\bar{L}\left(t\right)+\bar{I}\left(t,a\right)+\bar{C}\left(t,a\right)-q_{1}\left(t\right)\right)+\lambda_{1}h_{1}\left(t\right)-\lambda_{S}\left(t\right)p\bar{S}\left(t\right)+\lambda_{V}\left(t\right)p\bar{S}\left(t\right),$$

$$\frac{\partial H}{\partial h_{2}}=\lambda_{2}h_{2}\left(t\right)+\lambda_{S}\left(t\right)q_{2}\left(t\right)\bar{S}\left(t\right)-\lambda_{L}\left(t\right)q_{2}\left(t\right)\bar{S}\left(t\right),$$

$$\frac{\partial H}{\partial h_{3}}=\lambda_{2}h_{3}\left(t\right)-\lambda_{S}\left(t\right)\alpha \bar{R}\left(t\right)+\lambda_{R}\left(t\right)\alpha \bar{R}\left(t\right)+B_{1}\bar{R}\left(t\right).$$

So we get

$$\bar{h}\left(t\right)=\left({\bar{h}}_{1}\left(t\right),{\bar{h}}_{2}\left(t\right),{\bar{h}}_{3}\left(t\right)\right).$$

$$\begin{array}{lll} {\bar{h}}_{1}\left(t\right) & \;= & \frac{-B_{1}b\omega \left(\bar{N}\left(t\right)-m\bar{L}\left(t\right)-q_{1}\left(t\right)\right)+\lambda_{S}\left(t\right)p\bar{S}\left(t\right)-\lambda_{V}\left(t\right)p\bar{S}\left(t\right)}{\lambda_{1}},\\ {\bar{h}}_{2}\left(t\right) & \;= & \frac{-\lambda_{S}\left(t\right)q_{2}\left(t\right)\bar{S}\left(t\right)+\lambda_{L}\left(t\right)q_{2}\left(t\right)\bar{S}\left(t\right)}{\lambda_{2}},\\ {\bar{h}}_{3}\left(t\right) & \;= & \frac{\lambda_{S}\left(t\right)\alpha \bar{R}\left(t\right)-\lambda_{R}\left(t\right)\alpha \bar{R}\left(t\right)-B_{1}\bar{R}\left(t\right)}{\lambda_{3}}. \end{array}$$

$$\begin{array}{lll} h_{1}^{*}\left(t\right) & = & \;max\left(0,min\left({\bar{h}}_{1}\left(t\right),1\right)\right),\\ h_{2}^{*}\left(t\right) & = & \;max\left(0,min\left({\bar{h}}_{2}\left(t\right),1\right)\right),\\ h_{2}^{*}\left(t\right) & = & \;max\left(0,min\left({\bar{h}}_{3}\left(t\right),1\right)\right). \end{array}$$

**Fig. 4:**
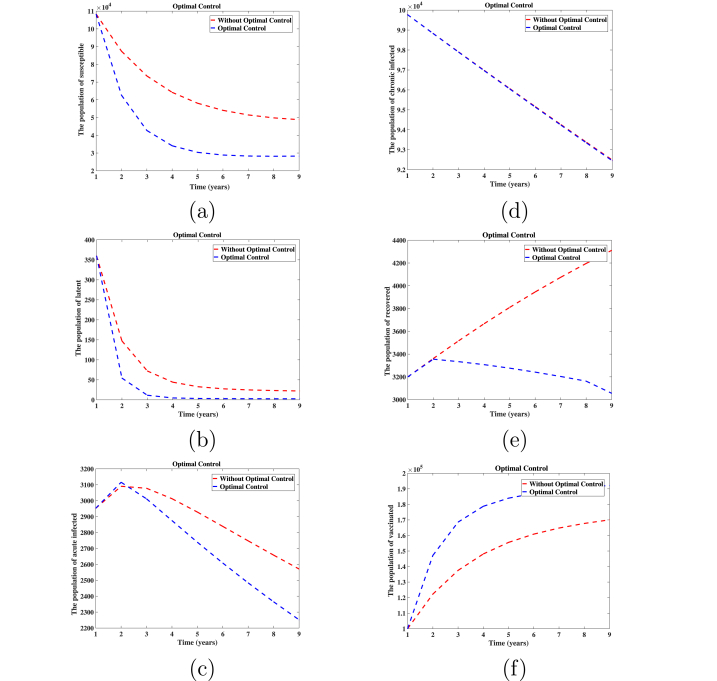
Comparison of changes in the number of controlled and non-controlled populations in six different populations.

To determine the optimal control and state system, we discretize time and employ the forward Runge-Kutta method for numerical integration of the system equations to obtain the system state at each time step. Subsequently, we utilize the backward fourth-order Runge-Kutta method to quantify the impact of various control measures. The algorithm provides an approximate approach to achieving optimal control. We use the parameter values obtained in Table 2 as the parameter values of the optimal control experiment, and the positive weight coefficients of the objective function are *A*_1_ = *A*_2_ = 170, *B*_1_ = 150, *B*_2_ = 60; they are explained in section 6, and we assume *λ*_1_ = *λ*_2_ = *λ*_3_ = 10. As shown in Fig. 4, the number of susceptible, latent, and acute cases that are subject to control measures exhibited a significant decrease. In contrast, there is a substantial increase in the number of vaccinated individuals. Additionally, the number of recovered cases decreases in tandem with the reduction in acute cases.

**Fig. 5:**
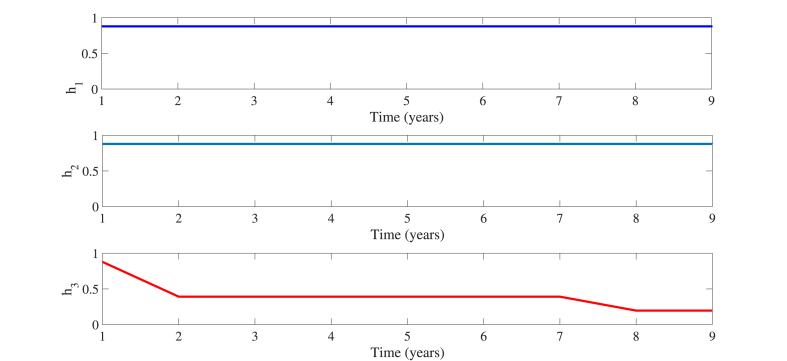
The three kinds of control *h*_1_, *h*_2_, *h*_3_ in system (5.1) correspond to the change curves respectively.

The results indicate that the simultaneous implementation of three control measures (*h*_1_, *h*_2_, *h*_3_) can maximize the optimal control effect and effectively curb the spread of HBV infection. To realistically, rationally, and efficiently manage the disease's progression, it is important to note that the intensity or efficacy of these measures, as implemented by governmental bodies or medical professionals, varies. The range of variation for the three control measures is [0, 1]. As shown in Fig. 5, the control intensity of each measure changes with time. *h*_1_(*t*) = 0.8791 and *h*_2_(*t*) = 0.8791 are more stable, *h*_3_(*t*) changes from 0.8971 to 0.1955. Ultimately, the effectiveness of optimal control has been demonstrated. Currently, it can only be observed from the optimal control strategy and Fig. 4 that the patient count can be reduced. However, the consumption and allocation of associated human, material, and other resources remain unclear. To enhance practical applicability, we will delve into a cost-effectiveness analysis in section 6.

## 6 Cost-effectiveness analysis

To identify the most cost-effective strategies for controlling HBV epidemics, we employ the incremental cost-effectiveness ratio (ICER) (Cantor & Ganiats, 1999; Li et al., 2012; Long et al., 2010; Okosun et al., 2013; Shen et al., 2018; Weinstein, 1996), a critical tool for evaluating the cost-effectiveness of different medical interventions. By clearly defining costs and benefits, comparing different interventions, and interpreting the ICER value, we can achieve a more comprehensive understanding of the economic implications of these interventions. Assume that *x* and *y* represent two different intervention strategies, *A*(*x*) and *A*(*y*) denote the total cases averted during the total period by the intervention strategies *x* and *y*, respectively,

$$\text{ ICER }=\frac{\text{ The difference between the cost of the intervention of }x\text{ and }y}{\text{ The difference between }A\left(x\right)\text{ and }A\left(y\right)\text{ interventions}}.$$

In recent years, we have obtained authentic data on acute patients across various age groups from the CDC (Screening and Testing for Hepatitis, 2023, p. 2024). To ensure the validity and reliability, we will focus on cases of acute patients who successfully avoided infection. Specifically, we will analyze the total number of cases that were prevented during time period T.

$$△I=\int_{0}^{T}\int_{0}^{A}I\left(t,a\right)dadt-\int_{0}^{T}\int_{0}^{A}I^{*}\left(t,a\right)dadt.$$

Based on section 5, *h*_1_ is the implementation of measures to control the vaccination of HBV vaccine. In the United States, when each susceptible person receives vaccination, in addition to paying the cost of three doses of medicine, they also need to pay the cost of manual vaccination of health care workers. It assumes that each person needs about 150 dollars (Success Story, 2024) for vaccination. *h*_2_ is the implementation of quarantine measures; during the quarantine period, there will be medical care and drug treatment; we assume that each infected person needs to spend about 170 dollars (Tantai et al., 2014) to implement isolation treatment. We assume that the cost of liver-related examinations per individual exceeds approximately *$*60, as these examinations primarily serve to detect reinfection rather than directly contribute to HBV treatment efficacy. Therefore, we do not solely on *h*_3_ as a control measure. The costs of implementing the various measures are shown in Table 4.

**Table 4:** The number of infections avoided and the cost of implementing different strategies.

Strategy	Infection averted	Total cost ($)
Strategy *a* (*h*_1_,*h*_3_)	618	117,457
Strategy *b* (*h*_1_)	1120	156,740
Strategy *c* (*h*_2_)	1404	224,563
Strategy *d* (*h*_2_,*h*_3_)	1416	297,306
Strategy *e* (*h*_1_,*h*_2_)	1737	521,150
Strategy *f* (*h*_1_,*h*_2_,*h*_3_)	2891	1,011,740

The specific calculation process of ICER value is in Appendix A. Different strategies and measures are listed according to the increasing trend of the number of people to avoid infection. In Table A.5, the ICER value of strategy (*d*) is significantly higher than that of strategy (*e*), indicating that the input cost of strategy (*d*) is higher than that of strategy (*e*), which consumes more limited resources and has poor implementation effect. Therefore, strategy (*d*) is excluded. Then rearrange according to the increasing effectiveness, again in the order of avoiding infection from the least to the most, and by recalculating ICER, we can get ICER(*a*) = 190.060, ICER(*b*) = 78.253, ICER(*c*) = 238.813, ICER(*e*) = 890.652, ICER(*f*) = 425.121. By comparison, it can be found that strategy (*e*) is more cost-consuming and less cost-effective and is excluded as an alternative. And then we continue to recalculate ICER, ICER(*a*) = 190.060, ICER(*b*) = 78.253, ICER(*c*) = 238.813, ICER(*f*) = 529.373. The strategy (*f*) has the largest ICER value and is therefore excluded. The remaining three implementation schemes of strategy (*a*), (*b*), and (*c*) are continued to calculate the ICER values of the three. ICER(*a*) = 190.060, ICER(*b*) = 78.253, ICER(*c*) = 238.813, strategy (*b*) has the smallest ICER value and is more cost-effective than strategy (*c*), excluding strategy (*c*). There are only two options left to compare, ICER(*a*) = 190.060, ICER(*b*) = 78.253; strategy (*b*) has the lowest ICER value and is more cost-effective.

Therefore, we have developed strategy (*b*), where vaccination against hepatitis B is the most cost-effective among the above six strategies.

## 7 Discussion and conclusion

This study examines acute hepatitis B cases in the United States from 2013 to 2021. The data shows the transmission dynamics of the hepatitis B virus are substantially influenced by age, the highest incidence of acute hepatitis B are patients aged 30–50 years old. Our model included infection-age structure to imitate the transmission of HBV, especially the age heterogeneity in horizontal and vertical (mother-to-child) transmission modes. The model also incorporates the incubation period and the vaccination status of the infected individuals. We found that most acute hepatitis B patients are aged 30–50 years old, which indicate that sexual contact, tattoos and injecting drugs could be the major transmission routes in the United States. Our model fitting results demonstrate that the incubation period of HBV is approximately six months, which is consistent with the official reports of the US CDC and WHO (Hepatitis B, 2024, Hepatitis B Basics, 2024, Hepatitis B Vaccine, 2024; Viral Hepatitis Surveillance, 2024).

To find the best intervention strategy, we perform a sensitivity analysis to identify key parameters that affect HBV transmission and control. The results reveal that parameters associated with mother-to-child transmission exhibited the least significant impact (Kim & Kim, 2018). In the United States, vertical transmission of HBV is the second most prevalent form of transmission, exceeded only by horizontal transmission. Given the most sensitive parameters observed in both acute and chronic cases, we have tested a few control measures, these include: (1) the administration of hepatitis B vaccines to infants and high-risk groups as soon as possible; (2) the isolation of acute patients and the implementation of drug treatment; (3) the requirement of regular liver examinations for recovered patients to prevent and treat HBV reinfection. Existing studies on hepatitis B, including those on optimal control, have not been combined with the actual burden of disease (Din et al., 2021; Jia et al., 2022; Jung et al., 2009; Kamyad et al., 2014; Khan et al., 2017; Laarabi et al., 2012; Lowden et al., 2014; Prosper et al., 2014), but our results are based on the CDC's specific burden on the hepatitis B epidemic in the United States, which has more detailed results and is more practical. We have used the Pontryagin Maximum Principle to find the most cost-effective control strategies. Considering resources are limited and each control measure cost money, our cost-effectiveness analysis focuses on six combination of control measures. We found that vaccination is the most cost-effective measure, early vaccination with a single-dose vaccine of US$50 is the most cost-effective control strategy.

WHO has set the goal of eliminating viral hepatitis by 2030, encompassing both developed and developing countries. The process of eliminating hepatitis B is complex and long-term. At present, WHO advocates for the widespread promotion and implementation of hepatitis B vaccination globally. The sterilization of medical devices and syringes is increasingly recognized as a critical and stringent requirement. With enhanced societal awareness and prevention of HBV infections, coupled with early intervention, effective control measures, and continuous advancements in medical standards, it is anticipated that the HBV epidemic can ultimately be controlled and eliminated.

Although the model and its parameters in this study are mainly based on data from the United States, its framework and methods are adaptable and can be applied to other regions by appropriate adjustments to reflect local conditions. Future studies should aim to validate and refine this model using data from different regions, particularly in developing countries where hepatitis B remains a major public health challenge. This will enhance the generalizability of the model and ensure its relevance in the global effort to combat hepatitis B.

In this study, we didn't consider the gender factor during the horizontal transmission patterns. Given that the sexual contact could be the major transmission routes, gender could be an important factor in HBV models. This area can be further studied in the future. Additionally, our model incorporates age heterogeneity by focusing on age at the time of infection to distinguish acute patients from chronic carriers, rather than considering chronological age. However, it remains challenging to derive parameters that are closely related to age dynamics based on real data. The study of chronological age may shed valuable insights into age-specific patterns and suggest implementing age-specific control measures. This area merits further exploration.

## CRediT authorship contribution statement

**Xuebing Chen:** Writing – original draft. **Yong Li:** Writing – review & editing, Funding acquisition, Formal analysis, Conceptualization. **Nurbek Azimaqin:** Investigation, Data curation. **Yan Wu:** Writing – review & editing, Formal analysis, Data curation. **Changlei Tan:** Writing – review & editing. **Xuyue Duan:** Writing – review & editing. **Yiyi Yuan:** Investigation.

## Ethics approval and consent to participate

The study design and methodology used only publicly available data sets. The open data sets we analyzed were identified prior to analysis and therefore do not require ethical approval.

## Funding

This research is supported by the 10.13039/501100001809National Natural Science Foundation of China (Nos. 12326335, 12326341), and the 10.13039/501100015310Natural Science Foundation of Xinjiang, China (No. 2022D01A198).

## Declaration of competing interest

The authors declare that they have no known competing financial interests or personal relationships that could have appeared to influence the work reported in this paper.

## Figures and Tables

**Table A.5:** ICER with different tactics

Strategy	Infection averted	Total cost ($)	ICER
Strategy *a* (*h*_1_,*h*_3_)	618	117,457	190.060
Strategy *b* (*h*_1_)	1120	156,740	78.253
Strategy *c* (*h*_2_)	1404	224,563	238.813
Strategy *d* (*h*_2_,*h*_3_)	1416	297,306	6061.917
Strategy *e* (*h*_1_,*h*_2_)	1737	521,150	697.333
Strategy *f* (*h*_1_,*h*_2_,*h*_3_)	2891	1,011,740	425.121

**Table A.6:** ICER with different tactics

Strategy	Infection averted	Total cost ($)	ICER
Strategy *a* (*h*_1_,*h*_3_)	618	117,457	190.060
Strategy *b* (*h*_1_)	1120	156,740	78.253
Strategy *c* (*h*_2_)	1404	224,563	238.813
Strategy *e* (*h*_1_,*h*_2_)	1737	521,150	890.652
Strategy *f* (*h*_1_,*h*_2_,*h*_3_)	2891	1,011,740	425.121

**Table A.7:** ICER with different tactics

Strategy	Infection averted	Total cost ($)	ICER
Strategy *a* (*h*_1_,*h*_3_)	618	117,457	190.060
Strategy *b* (*h*_1_)	1120	156,740	78.253
Strategy *c* (*h*_2_)	1404	224,563	238.813
Strategy *f* (*h*_1_,*h*_2_,*h*_3_)	2891	1,011,740	529.373

**Table A.8:** ICER with different tactics

Strategy	Infection averted	Total cost ($)	ICER
Strategy *a* (*h*_1_,*h*_3_)	618	117,457	190.060
Strategy *b* (*h*_1_)	1120	156,740	78.253
Strategy *c* (*h*_2_)	1404	224,563	238.813

**Table A.9:** ICER with different tactics

Strategy	Infection averted	Total cost ($)	ICER
Strategy *a* (*h*_1_,*h*_3_)	618	117,457	190.060
Strategy *b* (*h*_1_)	1120	156,740	78.253

## Appendix A ICER correlation calculation

(1) ICER's calculation is

$$\begin{array}{ll} & \mathrm{ICER}\left(a\right)=\frac{117457-0}{618-0}=190.060,\\ & \mathrm{ICER}\left(b\right)=\frac{156740-117457}{1120-618}=78.253,\\ & \mathrm{ICER}\left(c\right)=\frac{224563-156740}{1404-1120}=238.813,\\ & \mathrm{ICER}\left(d\right)=\frac{297306-224563}{1416-1404}=6061.917,\\ & \mathrm{ICER}\left(e\right)=\frac{521150-297306}{1737-1416}=697.333,\\ & \mathrm{ICER}\left(f\right)=\frac{1011740-521150}{2891-1737}=425.121. \end{array}$$

In Table A.5, the ICER value of strategy (*d*) is higher than that of other strategies. Therefore, strategy (*d*) is excluded. Continue to calculate ICER values for other policies.

(2) ICER's calculation is

$$\begin{array}{ll} & \mathrm{ICER}\left(a\right)=\frac{117457-0}{618-0}=190.060,\\ & \mathrm{ICER}\left(b\right)=\frac{156740-117457}{1120-618}=78.253,\\ & \mathrm{ICER}\left(c\right)=\frac{224563-156740}{1404-1120}=238.813,\\ & \mathrm{ICER}\left(e\right)=\frac{521150-224563}{1737-1404}=890.652,\\ & \mathrm{ICER}\left(f\right)=\frac{1011740-521150}{2891-1737}=425.121. \end{array}$$

In Table A.6, the ICER value of strategy (*e*) is higher than that of other strategies. Therefore, strategy (*e*) is excluded. Continue to calculate the ICER value of other policies.

(3) ICER's calculation is

$$\begin{array}{ll} & \mathrm{ICER}\left(a\right)=\frac{117457-0}{618-0}=190.060,\\ & \mathrm{ICER}\left(b\right)=\frac{156740-117457}{1120-618}=78.253,\\ & \mathrm{ICER}\left(c\right)=\frac{224563-156740}{1404-1120}=238.813,\\ & \mathrm{ICER}\left(f\right)=\frac{1011740-224563}{2891-1404}=529.373. \end{array}$$

In Table A.7, the ICER value of strategy (*f*) is higher than that of other strategies. Therefore, strategy (*f*) is excluded. Continue to calculate the ICER value of other policies.

(4) ICER's calculation is

$$\begin{array}{ll} & \mathrm{ICER}\left(a\right)=\frac{117457-0}{618-0}=190.060,\\ & \mathrm{ICER}\left(b\right)=\frac{156740-117457}{1120-618}=78.253,\\ & \mathrm{ICER}\left(c\right)=\frac{224563-156740}{1404-1120}=238.813. \end{array}$$

In Table A.8, the ICER value of strategy (*c*) is higher than that of the other two strategies, so strategy (*c*) is excluded. Continue to compare the ICER values of its strategies (*a*) and (*b*).

(5) ICER's calculation is

$$\begin{array}{ll} & \mathrm{ICER}\left(a\right)=\frac{117457-0}{618-0}=190.060,\\ & \mathrm{ICER}\left(b\right)=\frac{156740-117457}{1120-618}=78.253. \end{array}$$

Finally, in Table A.9, the ICER value of strategy (*a*) is significantly higher than that of strategy (*b*). Therefore, strategy (*a*) is excluded. Ultimately, strategy (*b*) (vaccination against hepatitis B) is the most cost-effective measure.
